# Hyperinsulinaemic Hypoglycaemia: Genetic Mechanisms, Diagnosis and Management

**DOI:** 10.4274/Jcrpe.821

**Published:** 2012-12-19

**Authors:** Zainaba Mohamed, Ved Bhushan Arya, Khalid Hussain

**Affiliations:** 1 University College London, Institue of Child Health, Developmental Endocrinology Research Clinical, Molecular Genetics Unit, London, United Kingdom

**Keywords:** Hyperinsulinism, hypoglycaemia in infancy, congenital hyperinsulinism, hyperinsulinaemic hypoglycaemia

## Abstract

Hyperinsulinaemic hypoglycaemia (HH) is characterized by unregulated insulin secretion from pancreatic β-cells. Untreated hypoglycaemia in infants can lead to seizures, developmental delay, and subsequent permanent brain injury. Early identification and meticulous managementof these patients is vital to prevent neurological insult. Mutations in eight different genes (ABCC8, KCNJ11, GLUD1, CGK, HADH, SLC16A1, HNF4A and UCP2) have been identified to date in patients with congenital forms of hyperinsulinism (CHI). The most severe forms of CHI are due to mutations in ABCC8 and KCJN11, which encode the two components of pancreatic β-cell ATP-sensitive potassium channel. Recent advancement in understanding the genetic aetiology, histological characterisation into focal and diffuse variety combined with improved imaging (such as fluorine 18 L-3, 4-dihydroxyphenylalanine positron emission tomography 18F-DOPA-PET scanning) and laparoscopic surgical techniques have greatly improved management. In adults, HH can be due to an insulinoma, pancreatogenous hypoglycaemic syndrome, post gastric-bypass surgery for morbid obesity as well as to mutations in insulin receptor gene. This review provides an overview of the molecular basis of CHI and outlines the clinical presentation, diagnostic criteria, and management of these patients.

**Conflict of interest:**None declared.

## INTRODUCTION

Hyperinsulinaemic hypoglycaemia (HH) represents a group of clinically, genetically, and morphologically heterogeneous disorders characterized by dysregulation of insulin secretion by pancreatic β-cells (1). It is the commonest cause of both transient and persistent states of hypoglycaemia posing significant risk of permanent brain damage (2,3,4). Insulin secretion from β-cells is precisely regulated by various mechanisms to maintain blood glucose values within the normal range (fasting blood glucose leSymptoms of hypoglycaemia can either be seen during fasting, or after a provels of 3.5–5.5 mmol/L). HH-unregulated insulin secretion drives glucose into insulin-sensitive tissues (skeletal muscle, liver and adipose tissue) and prevents the generation of alternative energy substrates (such as lactate and ketone bodies due to inhibition of glycogenolysis, gluconeogenesis, lipolysis and ketogenesis) thereby depriving the brain of glucose and ketone bodies. It is this metabolic milieu that results in hypoglycaemic brain injury (2).Clinically, HH can result in apneas, seizures, developmental delay, and learning disability (4). Hence, early identification and meticulous management of these patients is fundamental in preventing a neurological insult. HH can either be congenital hyperinsulinism (CHI) or secondary to certain risk factors like birth asphyxia, intra-uterine growth retardation (5), Rh isoimmunisation (6) and maternal diabetes mellitus or associated with various developmental syndromes like Beckwith-Wiedemann syndrome or metabolic conditions like congenital disorders of glycosylation (CDG syndromes) (7). In adults, an insulinoma accounts for most cases of HH. Other causes include noninsulinoma pancreatogenous hypoglycaemia syndrome (NIPHS), post gastric bypass surgery for morbid obesity and mutations in insulin receptor gene, which typically present with postprandial HH. The incidence of CHI can vary between 1 in 40 000–50 000 in the general population to 1 in 2500 in certain communities with high rates of consanguinity (8). The clinical presentation can be varied ranging from completely asymptomatic, pharmacologically responsive mild disease to severe disease unresponsive to medication needing surgical intervention (9).CHI are caused by genetic defects in key genes regulating insulin secretion. The genetic basis of CHI involves mutations in eight key genes (ABCC8, KCNJ11, GLUD1, GCK, HADH, SLC16A1, HNF4A and UCP2) (10,11,12,13,14,15,16,17) identified so far, which regulate insulin secretion from the β-cells. The most severe forms are due to recessive inactivating mutations in ABCC8 and KCJN11, which encode the two components of pancreatic β-cell ATP-sensitive potassium channel [encoding for the two proteins sulfonylurea receptor 1 (SUR1) and inward-rectifying potassium channel pore-forming (KIR6.2) of the pancreatic β-cell K_ATP_ channel, respectively]. Recessive forms of CHI, due to mutations in the shortchain hydroxyl-acyl-CoA dehydrogenase (HADH) are known to be rare (18). Dominant forms of CHI include activating mutations in encoding glutamate dehydrogenase (GLUD1), encoding glucokinase (GCK), encoding hepatocyte nuclear factor 4a (HNF4A) and encoding monocarboxylate transporter1 (SLC16A1) (12,15,16).Histological classification includes three major subgroups: diffuse, focal, and atypical, with the diffuse form inherited as autosomal recessive or dominant manner and the focal form being sporadic in inheritance. Pharmacologically unresponsive diffuse CHI may require a near-total pancreatectomy (with lifelong supplementation of pancreatic exocrine function andrisk of developing diabetes mellitus), whereas the focal form requires a focal lesionectomy [with advanced preoperativefluorine 18 L-3, 4-dihydroxyphenylalanine positron emission tomography / computed tomography (18F-DOPA-PET/CT) imaging aiding resection of the focal lesion] thereby curing the patient from the hypoglycaemia. In patients with atypical disease, the histological abnormalities may be diffuse with the coexistence of normal and abnormal islets (19). 

This review provides an overview of the clinical presentation, molecular basis, diagnostic tools, and management of HH with an emphasis on CHI.

**Clinical Presentation:**

HH is most commonly diagnosed in the newborn period; however, milder cases may be diagnosed either during infancy or childhood (2). Clinical symptoms are most severe in the newborn period. The infant may present with apnea, seizures and unresponsiveness, or the symptoms may be less severe and non-specific (poor feeding, irritability and lethargy). Newborns with CHI may be macrosomic due to hyperinsulinaemia in fetal life, particularly those who carry mutations in HNF4A (15). Some patients have hypertrophic cardiomyopathy and hepatomegaly probably reflecting fetal hyperinsulinaemia (20). HH may be associated with well-defined developmental syndromes (see reference table), the most common being Beckwith-Wiedemann syndrome. This is characterized by transient HI and pathognomonic physical signs which include exomphalos, hemihypertrophy, macroglossia, and transverse creases of the ear lobes (21). 

Symptoms of hypoglycaemia can either be seen during fasting, or after a protein-rich meal challenge or exercise. Some patients demonstrate marked sensitivity to dietary protein (especially leucine) (22). Patients with exercise-induced HH typically present with symptoms of hypoglycaemia within the 30-45 minutes following a period of intensive anaerobic exercise (23). The hypoglycaemia is often refractory to frequent enteral feeds, and intravenous glucose infusion rates in excess of 15-20 mg/kg/min are required to maintain a normoglycaemic state (24,25). Neonates usually require central venous access for delivering high concentrations of intravenous glucose. 

Adult-onset HH is usually caused by an insulinoma. The mean age at presentation is around 45 years; however, patients with insulinoma associated with type 1 multiple endocrine neoplasia usually present at younger ages. These patients usually develop hypoglycaemia symptoms after a fast but rarely have them postprandially. Patients with other conditions such as NIPHS, those who underwent gastric bypass surgery for severe obesity, and those with mutations in the insulin-receptor gene can also have postprandial hypoglycaemia (26,27,28,29).

**Diagnosis of HH:**

Early recognition of HH is vital in preventing neuroglycopaenic brain injury. The diagnosis of HH is based on clinical presentation and on detection of its biochemical markers at the time of hypoglycaemia. Plasma insulin levels may not be dramatically elevated in HH; rather, there is inadequate suppression of insulin at low blood glucose concentrations (2). Insulin release is pulsatile and measurement of C-peptide might be more helpful in some patients where the diagnosis is in doubt. 

The diagnosis is most frequently based on evidence of excessive insulin action. The characteristic metabolic profile is hypoketonaemic, hypofattyacidaemic hypoglycaemia arising from the anabolic effects of insulin. A high glucose requirement (>8 mg/kg min, normal range 4-6 mg/kg min) to maintain normoglycaemia is a strong indicative measure of a defect in regulation of basal insulin secretion. In difficult to diagnose cases, supportive evidence can be provided by decreased serum levels of insulin-like growth factor-binding protein 1 (IGFBP-1) (as insulin suppresses the transcription of IGFBP-1 gene), a positive glycemic response to intramuscular/ intravenous glucagon at the time of hypoglycaemia (a clear increment in blood glucose of >1.5 mmol/L despite severe hypoglycaemia), and a positive glycemic response to octreotide (30,31). 

Additional tests for specific forms of CHI include measuring the plasma ammonia level (12), performing a leucine provocation test (GDH-HI), assessment of the plasma acylcarnitine profile (elevated 3-hydroxybutyryl-carnitine) and urine organic acids (3-hydroxyglutarate in urine; HADH-CHI) (14). Performing an exercise provocation test and/or a pyruvate load test (SLC16A1-CHI) may also be helpful (23).In adults with HH, a 72-h fast will detect 99% of insulinoma, which is the most common cause of HH in adults (32). Patients with postprandial HH do not exhibit symptoms after fasting tests, but hypoglycaemia can be detected by an oral glucose tolerance test (OGTT) or by a mixed-meal provocation test. No consensus exists on the best method with which to investigate postprandial HH. The OGTT, in particular, is often followed by a physiological dip in blood glucose level, which might lead to misdiagnosis. However, one can distinguish between pathological postprandial HH and ‘reactive’ hypoglycaemia by looking for corresponding biochemical evidence of endogenous hyperinsulinaemia and symptoms of neuroglycopenia during a hypoglycaemic episode. Elevated plasma insulin levels with undetectable C-peptide levels indicate exogenous insulin administration. Measurement of sulfonylureas in plasma and urine is recommended in all patients in whom the cause of HH is unclear, to rule out factitious hypoglycaemia due to administration of sulfonylureas.

**Aetiology of HH**

**(1) Transient HH**

When HH resolves spontaneously within a few days or a few weeks, a diagnosis of transient HH is made. Maternal diabetes mellitus (gestational or insulin-dependent), intra-uterine growth restriction, perinatal asphyxia, erythroblastosis fetalis, maternal administration of drugs such as sulphonylureas, and intravenous glucose infusions during labour tend to cause a transient form of HH. There have been case reports of transient HH which were not associated with any of the above risk factors (33). Transient HH can be protracted in some patients with intrauterine growth restriction and asphyxia, requiring treatment with diazoxide (34). The underlying mechanism for transient HH remains unclear.

**Molecular mechanisms of congenital forms of HH:The roleof pancreatic β-cell function K_ATP_ channels in regulating insulin secretion**

K_ATP_ channels in pancreatic β-cells were first described to play an important role in insulin secretion over 20 years ago (35). They regulate the flux of K ions across cell membranes and thereby link cell metabolism to electrical activity. Glucose phosphorylation by GCK is the rate-limiting step that controls glucose-regulated insulin secretion. Further metabolism of glucose-6-phosphate via glycolysis generates ATP causing an increase in the intracellular phosphate potential (ATP/ADP ratio), which inhibits the activity of an ATP-sensitive K+ channel leading to closure of the K_ATP_ channels resulting in membrane depolarization. This subsequently activates voltage-dependent calcium channels leading to an influx of Ca which in turn triggers the secretory granule to release insulin (36,37,38,39). 

**Figure 1:**
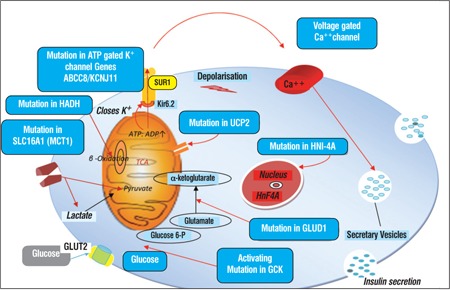
Schematic representation of the known causes of HH inthe pancreatic β-cell

The K_ATP_ channel is a hetero-octameric complex composed of four Kir6.2 subunits and four high-affinity SUR1 subunits (39). This channel has a role in linking glucose metabolism in theβ-cell to insulin secretion. Glucose metabolism in β-cells raises the intracytosolic ATP/ADP ratio which inhibits the plasma membrane SUR1, thus closing the K_ATP_ channel. This closure leads to cell membrane depolarization, Ca2+ influx via the voltage-gated Ca channels and release of insulin from storage granules (Figure 1).

**(2) Congenital Hyperinsulinism (CHI):**

Congenital hyperinsulinism is a group of genetic disorders comprising abnormalities in key genes involved in regulating insulin secretion. Only 50% of monogenic forms of CHI have been identified to date. As shown in Table 1, these are classified as:

**(a) ChannelopathiesPancreatic β-cell K_ATP_ channel defects:**

The most common and most severe form of CHI results from recessive inactivating mutations in ABCC8 and KCNJ11 genes (10,11). Loss-of-function mutations in these genes deregulate the precise mechanism by which glucose metabolism controls insulin secretion. The β-cell plasma membrane is constantly depolarized, causing Ca2+ influx and insulin secretion. The molecular basis of recessive inactivating ABCC8 and KCNJ11 mutations involves multiple defects in K_ATP_ channel biogenesis and turnover, in channel trafficking from the ER and Golgi apparatus to the plasma membrane, and in alterations of channels in response to both nucleotide regulation and open state frequency (40,41,42,43). 

As diazoxide is a K_ATP_ channel agonist, the patients are usually diazoxide-unresponsive when the defect in ABCC8 or KCNJ11 abolishes the function of this channel. Dominant inactivating mutations of ABCC8 and KCNJ11 usually cause milder forms of CHI although recently, medically unresponsive forms have been reported (44).

**Histological Subtypes of HH:**

Defects in K_ATP_ channel lead to two clinically indistinguishable histopathological subtypes of HH - focal and diffuse. Focal HH is sporadic, while diffuse HH is autosomal recessively inherited or more rarely dominantly inherited. In diffuse HH, β-cells throughout the pancreas are functionally abnormal and have characteristic enlarged nuclei in about 2%-5% of cells. Focal HI lesions are usually <10 mm in diameter and are characterized by the presence of a confluent proliferation of islet-cell clusters (focal adenomatosis) (45). The abnormal or enlarged islet cell nuclei are often observed within the lesion but are not seen outside of the focal lesion where the islets exhibit small nuclei and less cytoplasm, with evidence of low proinsulin synthesis. Intraoperative frozen sections are essential, not only to confirm the diagnosis based on imaging but also to determine whether the suspected area effectively corresponds to a focal lesion and is completely resected. 

In GCK-HI, descriptions of islet cell morphology vary, with normal-appearing islets in some cases and enlarged islet size in others. Histological studies have described diffuse islet cell “hyperplasia” in Beckwith-Wiedemann syndrome. Recently, a new histological form of HH has been described that is characterized by a morphological mosaicism. The abnormal hyperfunctional islets of this form are most often confined to a few adjacent lobules with possible cure by limited pancreatectomy (46).

**1. Focal**

Focal forms can either involve a small area (approximately 2.5 to 10 mm in diameter) of the pancreas or rarely very large areas. The focal lesions are made of large endocrine cells which have a large cytoplasm and nuclei that are 3-5 times the size of nearby acinar nuclei. The area of abnormal pancreatic development is multilobular and can have satellites in the nearby pancreatic tissue that necessitate intraoperative margins analysis to ensure complete excision and avoid recurrence. The focal lesions can sometimes be deeply embedded within the pancreatic tissue. Focal disease is always sporadic and has a distinctive genetic aetiology involving two independent events – inheritance of a paternal mutation in ABCC8 or KCNJ11 and somatic loss of the maternal 11p allele (11p15.1 to 11p15.5) involving the ABCC8 and KCNJ11 region within the focal lesion (47). The maternal allele loss unmasks the paternally inherited K_ATP_ channel mutation in addition to an imbalance in the imprinted genes in this region (maternally expressed tumour suppressor genes H19 and CDKN1C, and the paternally expressed growth factor IGF2). An imbalance of the 11p15 region leads to increased proliferation of β-cells evolving into a focal adenomatous hyperplasia.

**2. Diffuse**

The diffuse form affects the whole of pancreas with variable involvement of the islets. The islet pattern is preserved, but it contains very active β-cells with very abundant cytoplasm and highly abnormal nuclei 3-4 times larger than normal size. The most common causes of diffuse CHI are the recessive and dominant mutations in ABCC8 and KCNJ11 (10,11). Neither clinical nor biological data can be used to differentiate the focal from the diffuse forms. However, the recently introduced noninvasive imaging using PET with 18F-DOPA is valuable in distinguishing these two forms (48,49).

**3. Atypical**

Atypical forms remain poorly defined. The pattern of pancreatic involvement may be of the diffuse type but may be confined to one large area of the pancreas. The genetic basis of these atypical forms is yet to be clearly elucidated but some are explained by chromosomal mosaicism (50). A new form of HI that is characterized by a morphological mosaicism and a particular histology of limited islet nuclear enlargement (LINE) has been reported (46). The abnormal hyperfunctional islets of this form are most often confined to a few adjacent lobules. Over a 25-year period, 217 cases of operated persistent HI were reviewed, of which 16 cases (7.4%) did not fit with the usual types. They displayed peculiar pancreatic morphologies and had a distinct clinical presentation, with normal birth weight in the majority of the cases, a late onset of HH and a relative sensitivity to diazoxide. Furthermore, when genetic analyses were performed in these infants (n=13), genomic mutations of ABCC8, KCNJ11, and GCK were ruled out, and no similar peculiar pathologies were found in cases with similar mutations. Intraoperative recognition and diagnosis of this particular form of HI can often be cured by a limited pancreatectomy (46).

**(b) Enzyme Defects**

**I.Hyperinsulinism-Hyperammonaemia Syndrome (HI/HA):**

HI/HA syndrome, the second most common form of CHI, is caused by activating mutations in the GLUD1 gene, which encodes for the intramitochondrial enzyme glutamate dehydrogenase (GDH) (12,51,52,53,54,55,56). GDH activity is regulated by allosteric inhibitors (GTP) and activators (ADP and leucine). Mutations in GLUD1 lead to a gain of enzyme function by reducing its sensitivity to allosteric inhibition by high-energy phosphates such as GTP and ATP and allowing activation by the amino acid leucine.

GDH catalyzes the oxidative deamination of glutamate to α-ketoglutarate and ammonia using NAD+ and/or NADP+ as co-factors and is widely distributed at high levels in the pancreas, liver, brain, kidney, heart, and lungs. In pancreatic β-cells, α-ketoglutarate enters the Krebs cycle and increases the ATP/ADP ratio, thereby triggering the insulin release through inhibition of K_ATP_ channels. Increased GDH activity in liver may lead to HA because of excessive ammonia production and impaired urea cycle activity although this is not confirmed. Some recent animal studies suggest the role of renal ammoniagenesis due to activation of GDH as a source of HA in these patients (57). Sporadic (80%) and dominantly inherited (20%) mutations have been reported in the GTP-inhibitory allosteric binding site or in an antenna region of the enzyme.

Affected children have recurrent postprandial hypoglycaemia following protein-rich meals together with persistently elevated plasma ammonia. These patients do not typically present with hypoglycaemia at birth and frequently remain undiagnosed until several months of age and may present with seizures. Plasma ammonia levels are typically 2–5 times the upper limit of normal and stable with fasting and protein meals. There are patients described with HI/HA syndrome with persistently normal serum ammonia. HA is usually asymptomatic and does not warrant treatment. The hypoglycaemia in patients with HI/HA syndrome is usually diazoxide-responsive (58).

**II. Hydroxyacyl Coenzyme A Dehydrogenase and Hyperinsulinism (HADH and HI)**

Mutations in HADH, the gene encoding the mitochondrial enzyme L-3 HADH, are a rare cause of recessively inherited HH. HADH catalyzes the penultimate step in the β-oxidation of fatty acids (the nicotinamide adenine dinucleotide (NAD+)-dependent dehydrogenation of L-3 HADH to the corresponding 3-ketoacyl-coenzyme A.

HADH is highly expressed in pancreatic β-cells, suggesting it has an important role in insulin secretion. HADH expression is regulated by transcription factors (such Foxa2) that are crucial for proper β-cell differentiation and function. β-cell-specific Foxa2 knockout mice show a 3-fold down-regulation of HADH and severe HH (59). Suppression of HADH activity using small interfering RNA caused a significant increase in basal insulin secretion compared with untreated cells. The addition of diazoxide did not alter the enhanced basal insulin secretion caused by HADH suppression, indicating that HADH functions through K_ATP_-independent pathway to regulate insulin secretion (60,61).

The precise mechanism behind dysregulated insulin secretion in patients with HADH mutations is not well understood but might involve an interaction between HADH and GDH as protein sensitivity, which is a feature of gain-of-function mutations in GDH and which has also been demonstrated in patients with HADH mutations. This finding has been confirmed in HADH knockout mice (62). Direct protein-protein interactions between GDH and HADH have been demonstrated in control human lymphoblasts, which are lost in patients with HADH mutations causing leucine-induced HH (63). It is likely that HADH mutations cause leucine sensitivity and dysregulated insulin secretion via a novel pathway not involving GTP regulation of GDH.

The clinical phenotype of HADH mutations varies from severe neonatal HH to mild late-onset HH. Abnormal acylcarnitine metabolites (raised plasma hydroxybutyrylcarnitine and urinary 3-hydroxyglutarate levels) might give a clue to the diagnosis in some patients (14). In contrast to other defects in fatty acid oxidation, children with HADH mutation have no signs of hepatic dysfunction, cardiomyopathy, or effects on skeletal muscle. All patients reported so far have responded to diazoxide. Genetic analysis of HADH gene is recommended in patients with diazoxide-responsive HH from consanguineous families, who are negative for mutations in the K_ATP_ channels (64).

**III. Exercise-Induced Hyperinsulinism (EIHI) (SLC16A1)**

EIHI is a dominantly inherited condition due to mutations in the promoter region of SLC16A1 gene, leading to increased expression of the plasma membrane monocarboxylate transporter 1 (MCT1) in the β-cell (16). Under normal conditions, expression of MCT1 in the pancreatic β-cell is very low, which minimizes the effects of pyruvate and lactate on insulin secretion. miR-29a and miR-29b (microRNAs) have been shown to contribute to pancreatic β-cell-specific silencing of MCT1 (65).

The increased level of MCT1 due to promoter-activating mutations in SLC16A1 allows the circulating pyruvate/lactate to enter β-cells, where it acts as a substrate for mitochondrial oxidation leading to an increased cytosolic ATP/ADP ratio. This triggers insulin release despite the absence of elevated blood glucose levels, resulting in hypoglycaemia. Affected patients become hypoglycaemic typically 30 to 45 minutes after a period of intensive anaerobic exercise due to accumulation of lactate and pyruvate (16).

**IV. Glucokinase- İnduced Hyperinsulinism (HI)**

Activating glucokinase (GCK) mutations are a rare cause of medically responsive CHI (66). Glucose phosphorylation by GCK is the rate-limiting step that controls glucose-regulated insulin secretion. Activating mutations lead to a left shift of the glucose dependency curve resulting in lower threshold for glucose-stimulated insulin release. Glucokinase has a similar role as glucose sensor in other body cells such as entero-endocrine cells, hepatocytes, and hypothalamic neurons (67).

The activating GCK mutations are inherited in an autosomal dominant manner with a highly variable phenotype even within families with the same GCK mutation. GCK-CHI can either present during the neonatal period or at a much later stage, even as late as adulthood. The majority of GCK-CHI patients are diazoxide-responsive, but occasional severe phenotypes require more intensive management including octreotide and subtotal pancreatectomy (68). Diazoxide-responsive CHI patients, negative for K_ATP_ mutations, should be considered for GCK genetic testing. Although GCK-CHI is known to be of low prevalence, if an activating GCK mutation is identified in the family, other members should be screened to detect asymptomatic or mild symptomatic CHI (69).

**V. Mutations in the Mitochondrial Uncoupling Protein 2**

**(UCP2) Gene and Hyperinsulinaemic Hypoglycaemia (HH)**

UCP2 induces a regulated leak of protons across the inner mitochondrial membrane and uncouples mitochondrial oxidative metabolism from ATP synthesis. Consequently, cell ATP content decreases, as well as insulin secretion. UCP2 over-expression in rat-isolated pancreatic islet cells decreases the ATP content and inhibits glucose-stimulated insulin secretion (17). Conversely, UCP2 knockout mice exhibit HH. UCP2 loss-of-function mutations were recently reported in two unrelated children with neonatal-onset congenital HH and hypoglycaemia which were diazoxide-responsive (17).**(c) Transcription Factor Defects**

**HNF4A and HH**

Mutations in HNF4A are one of the less common causes of HH, which can either be transient or persistent. HNF4A gene encodes for the transcription factor hepatocyte nuclear factor 4 alpha (HNF-4α), which belongs to the nuclear hormone receptor superfamily and is required in the pancreatic β-cell for regulation of the pathway of insulin secretion. Loss-of-function HNF4A mutations cause maturity-onset diabetes of the young type 1 (MODY1), which is characterized by progressive β-cell destruction and failure of glucose-induced insulin secretion (70,71).

HNF-4α binds to the promoters of 11% of the pancreatic islets genes, and it is quite likely that HNF4α deficiency probably exhibits its phenotype via abnormal gene expression of one or more of these target genes. The possible mechanism behind HH in HNF4A mutations either might be reduction in expression of the K channel subunit Kir6.2 and/or reduction in the expression of nuclear receptor peroxisome proliferator-activated receptor α (PPARα). PPARα shifts energy metabolism in cells towards fatty acid oxidation (FAO) in response to starvation. Isolated islets from PPARα null mice were noticed to have a 44% reduction in FAO and enhanced glucose-induced insulin secretion (72,73).

The clinical phenotype ranges from macrosomia to mild transient hypoglycaemia not requiring pharmacological treatment or to persistent HH treated with diazoxide for up to 8 years. Only a minority of HNF4A mutation carriers have been reported to develop HH. Mutations may arise de novo, and parents of HNF4A-mutation children might not have diabetes. Hence, the absence of a history of diabetes in the parents should not preclude sequencing of the HNF4A gene (71).

**(3) Postprandial forms of HH**

Inappropriate insulin secretion in response to a meal can lead to hypoglycaemia within a few hours of meal ingestion (postprandial HH). In children, the most common cause of postprandial HH is ‘dumping’ syndrome seen in infants who have undergone Nissen’s fundoplication (74). Precipitous emptying of hyperosmolar carbohydrate-containing solutions into the small bowel shifts fluid into the bowel lumen,which results in hypovolaemia, rapid glucose absorption, hyperglycaemia, and reactive hypoglycaemia. It has been noted that children with postprandial HH after Nissen’s fundoplication have abnormally exaggerated secretion of glucagon-like peptide-1 (GLP-1), which may contribute to the exaggerated insulin surge and resultant hypoglycaemia (75).

Patients with postprandial HH do not exhibit symptoms after fasting tests, but hypoglycaemia can be made apparent by an OGTT or by a mixed-meal provocation test. No consensus exists on the best method to investigate postprandial HH. The OGTT, in particular, is often followed by a physiological dip in blood glucose level, which might lead to misdiagnosis. However, one can distinguish between pathological postprandial HH and ‘reactive’ hypoglycaemia by looking for corresponding biochemical evidence of endogenous hyperinsulinaemia and symptoms of neuroglycopenia during a hypoglycaemic episode (either spontaneous or following a provocation test). A decrease of >6 mmol/L between peak and nadir glucose has been proposed as a diagnostic criterion for dumping syndrome (76).

Most other presentations of postprandial HH have been reported in adults. Postprandial HH has been described in patients with insulin autoimmune syndrome. NIPHS (77) has been described in patients who have undergone gastric bypass surgery for morbid obesity (78) and in carriers of mutations in the insulin-receptor gene (26).

**(4) Other Causes of HH**

(Table 2)The term Munchausen syndrome by proxy (MSP) describes a form of child abuse involving the mother's, a parent's, or another guardian's falsifying illness in a child. Diagnosis is difficult because caregivers are adept at deceiving medical and mental health professionals. Cases of chronic surreptitious administration of insulin or antidiabetic drugs such as sulfonamides to children and adults have been reported, with some being treated with pancreatectomy to control hypoglycaemia (79, 80).

**Figure 2:**
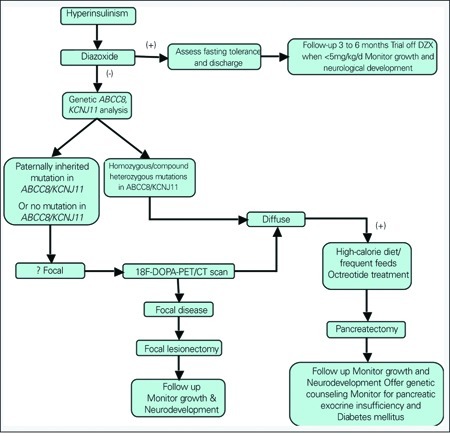
Outline of suggested management pathway

**Management** (Figure 2):

**1) Immediate Management**

Prompt diagnosis with aggressive early intervention to prevent hypoglycaemia remains the mainstay of treatment required to avert irreversible brain damage (4,81). Due to the hypoketonaemic, hypofattyacidaemic hypoglycaemia arising from the anabolic effects of insulin preventing the generation of alternative brain fuel, it is recommended that a higher threshold of blood glucose concentration be used to intervene and that this level is maintained (operational threshold for intervention in HH is 3.5 mmol/L, below which will lead to neuroglycopenia) (82). This often requires the insertion of a central venous catheter to deliver concentrated solutions of glucose intravenously.

A combination of oral feeds with a glucose polymer (such as SOS, Maxijul or Polycal) and intravenous fluids can be used to meet the carbohydrate requirement in these infants. It is vital to determine the minimum glucose infusion rate required to maintain normoglycaemia both for diagnostic and management purposes (a rate of >10 mg/kg/min is a specific diagnostic marker for HH) (2). Glucose requirement may exceed the glucose infusion tolerance needing a concomitant IV glucose infusion with enteral feeding. The severity of hypoglycaemia or the rate of glucose required to maintain normoglycaemia cannot accurately predict the form of hypoglycaemia: whether it is transient, genetic, focal, or diffuse.

**Emergency Drug Treatment:**

**Glucagon:**

Intramuscular glucagon (0.5-1 mg) can be used in an emergency situation where venous access is difficult to obtain. Continuous subcutaneous or intravenous glucagon infusion can be initiated for refractory hypoglycaemia despite high glucose infusion rate during acute management. Glucagon can be administered alone or in combination with octreotide. Glucagon improves blood glucose concentration by immediate release of glycogen stores from the liver and stimulating gluconeogenesis, ketogenesis, and lipolysis (83). In high doses, glucagon can cause paradoxical insulin secretion, implying that infants receiving glucagon bolus should receive intravenous glucose infusion to prevent rebound hypoglycaemia. Glucagon has no role in long-term management of HH (84).

**2) Further management**

This involves assessing the response to different medical therapies (83). When there is no improvement in the first few days (transient HI e.g. gestational diabetes), specific treatment outlined for HI should be initiated. It is important to assess the response to each medication before moving onto the next step.

**Diazoxide:**

Diazoxide is the first-line drug for management of HH. It acts by opening the K_ATP_ channels via binding to the intact SUR1 component, thereby preventing glucose-stimulated insulin secretion. Figure 1 shows the management cascade and outlines the medical therapies used. Fluid retention and hypertrichosis are the most common side effects of diazoxide. In newborns, it is used in conjunction with chlorothiazide, a diuretic, to overcome the adverse effects of fluid retention (83). In older children, hypertrichosis can sometimes be marked and distressing but will be reversible after its withdrawal (85). Diazoxide is effective in virtually all forms of HH except in those due to recessive (and some dominant) inactivating mutations in ABCC8 and KCNJ11 and in patients with focal CHI. Diazoxide responsiveness is determined by a) appropriate fasting tolerance for age; b) feed volume and frequency normal for age; c) normal blood sugar levels at the end of the fast. Octreotide must be tried before considering surgery in case of diazoxide-unresponsiveness (86). In these patients, further investigations (genetic analysis or mutations in ABCC8/KCNJ11 and 18F-DOPA-PET/CT scan) are essential to differentiate focal from diffuse disease as the surgical approaches are radically different.

**Somatostatin Analogue: Octreotide**

Octreotide is the second-line drug for HH and is given as 6-8 hourly by subcutaneous injections. The starting dose is 5-10μg/kg/day and the dose can be increased according to response up to 15-40 μg/kg/day. Tachyphylaxis leads to a rapid decrease in the response to octreotide 24-48 hours after treatment initiation. Hence, response can be assessed only 2 days after the initiation of a new dose. The criteria for responsiveness to octreotide are the same as for diazoxide. The common side effects include vomiting and/or diarrhea and abdominal distension, which resolve spontaneously within a week of initiation. However, fatal necrotizing enterocolitis has also been reported in some neonates, which warrants careful monitoring of neonates on octreotide treatment (87). Gallbladder sludge or stones are rare long-term complications which necessitate serial ultrasound screening. Long-acting somatostatin analogues are being studied in different HI centers to investigate their effectiveness and their impact on the patients’ quality of life (88, 89).

**Response to Medical Therapy:**

Spontaneous clinical recovery can occur in some patients relatively early (within several months) or later (several years). Most patients outgrow the dose of diazoxide and octreotide during childhood and can be assessed by controlled fast off these medications. Once they are on a very small dose, the treatment can be stopped to assess resolution of HH. However, some patients require continuous adjustment of the dose due to increase in size as they grow. Recurrence of hypoglycaemia may occur if this adjustment is not made. Such patients may continue to require diazoxide or octreotide for decades (90).

**Diazoxide-Unresponsive CHI:**

The management of patients who are diazoxide unresponsive has radically changed in the last few years due to advances in molecular genetics, radiological imaging techniques and laparoscopic surgery. In diazoxide-unresponsive patients, it is essential to have rapid genetic analysis for mutations in ABCC8/KCNJ11 and/or 18F-DOPA-PET/CT scan to plan subsequent treatment (91). Patients with genetically confirmed diffuse disease do not require further imaging studies. Mutations in ABCC8 and KCNJ11 allow identification of the majority of patients with diffuse disease (homozygous or compound heterozygous mutations in ABCC8 and KCNJ11).Patients with a paternally inherited mutation in ABCC8 or KCNJ11 (or those with no mutations in these genes) potentially can have a focal disease and thus will require further imaging studies with 18F-DOPA-PET scan to differentiate focal from diffuse disease. In addition, it allows precise pre-operative localization of the focal lesion. Nearly 50% of diazoxide-unresponsive patients have focal disease. 18F-DOPA-PET/CT-scan localization and limited surgical removal leads to complete cure of the hypoglycaemia. In contrast, patients with diazoxide-unresponsive diffuse disease may require a near-total pancreatectomy which will have lifelong implications (high risk of diabetes mellitus, pancreatic exocrine insufficiency) (92, 93).

**Fluorine 18 L-3, 4-Dihydroxyphenylalanine Positron Emission Tomography (18F-DOPA-PET):**

This is a novel imaging technique which helps in thepre-operative localization of focal lesions (48). The principle of 18F-DOPA-PET scan is based on the tissue uptake of L-DOPA. Pancreatic islets are able to uptake L-DOPA and convert it to dopamine through DOPA decarboxylase, so that DOPA hyperfunctional activity can be traced. The uptake of the positron emitting tracer 18F-DOPA-PET is increased in ß-cells with a high rate of insulin synthesis and secretion compared to unaffected areas allowing visualization of the focal lesion. The sensitivity for detecting focal lesions varies between 88 and 94% with an accuracy of 100% (94). Therefore, this technique plays an important role in indicating and determining the type of surgery (partial pancreatectomy in case of a focal form or subtotal pancreatectomy for diffuse form) (95,96,97,98,99).

**Surgical Management of CHI**

The focal form of the disease requires a limited pancreatectomy, whereas diffuse disease will require a near-total pancreatectomy (45). In the surgery for excision in patients with the focal form, per-operative histology will search for abnormal cells at the margin. Additional resections until margins are clear may need to be performed. The patient can be cured from the hypoglycaemia if the focal lesion is completely excised. Diffuse HI requires near-total pancreatectomy (95-98% of the pancreas) leaving just the small triangle of pancreatic tissue between the duodenum and the common bile duct. Disturbance in glucose homeostasis may persist (50%) but is more manageable than before surgery. The use of laparoscopy represents a new approach to the diagnosis and management of infants with CHI (100). Near-total pancreatectomy is associated with a high incidence of diabetes mellitus and pancreatic exocrine insufficiency, hence reserved for those severe cases where all medical therapy has failed (93,101).

**Medical Management of Diazoxide-Unresponsive Diffuse CHI**

In patients who are unresponsive or partially responsive HI, it is paramount to provide high amounts of glucose to maintain normoglycaemia. This can be achieved by frequent glucose-enriched oral feedings and frequent or continuous enteral feedings. The principle of this treatment is based on the fact that the hypoglycaemia in some patients gradually gets milder over time. Some infants with diazoxide-unresponsive diffuse disease may be managed with long-term subcutaneous octreotide injections in combination with frequent feedings (88). A gastrostomy is recommended in these patients to allow the delivery of frequent bolus and continuous overnight feeds. Successful treatment with long-acting octreotide has been reported in ten patients, with an average follow-up of 17 months. Ongoing studies on long-acting somatostatin analogues investigate their effectiveness and their impact on the patient’s quality of life (102).

**Summary**

HH is an important cause of hypoglycaemia in childhood caused by unregulated insulin secretion by β-cells. Hence, early identification and meticulous management of these patients is vital to prevent neurological insult. Improved understanding of the genetic mechanisms leading to the CHI has begun to unfold the heterogeneity seen with this condition providing new insights into the mechanisms involved in insulin secretion. Recent advances in the fields of molecular genetics coupled with imaging techniques (18F-DOPA-PET scanning) and laparoscopic surgery have improved the clinical care of infants with CHI. 

## Figures and Tables

**Table 1:**
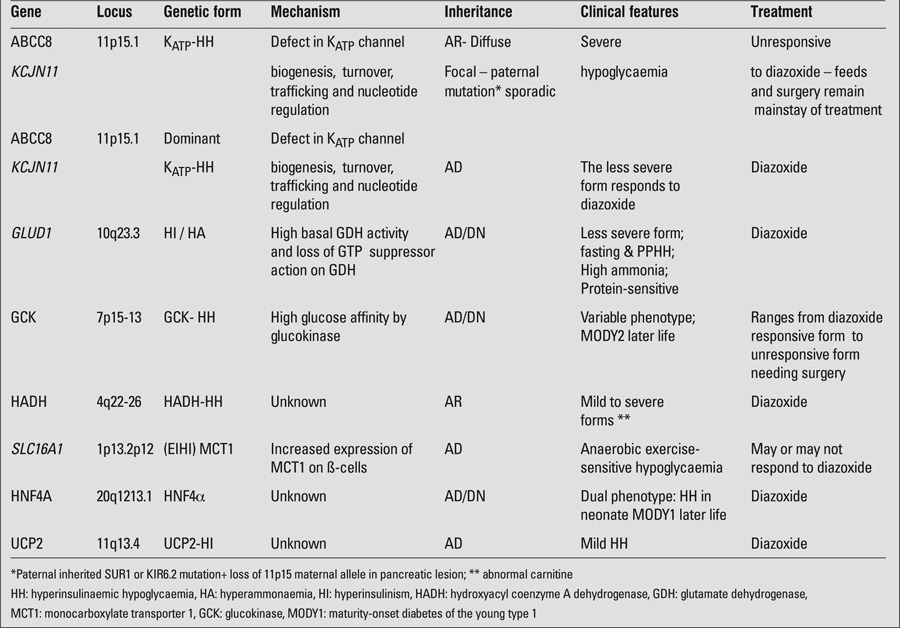
Genetic classification of congenital hyperinsulinism

**Table 2:**
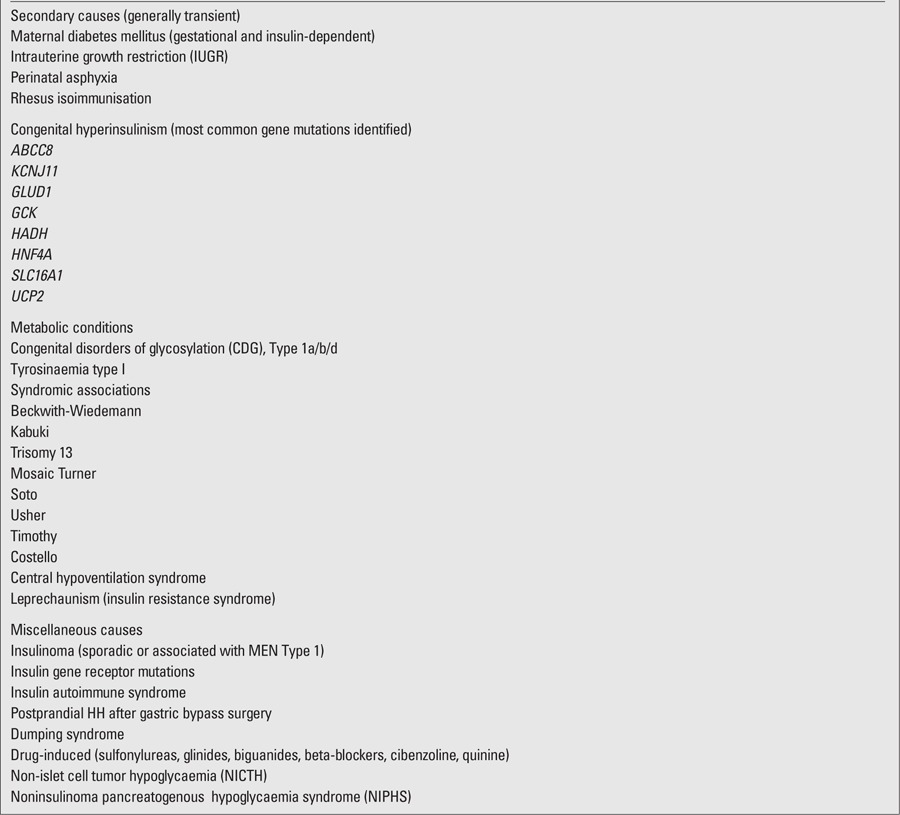
Causes of hyperinsulinaemichypoglycaemia (HH)
